# Changes in refraction after cataract phacoemulsification surgery

**DOI:** 10.1007/s10792-022-02550-9

**Published:** 2022-10-12

**Authors:** Natalie Si-Yi Lee, Keith Ong

**Affiliations:** 1 Faculty of Medicine, University of New South Wales, Sydney, Australia; 2 Northern Clinical School, University of Sydney, Sydney, Australia; 3 Chatswood Private Hospital, Sydney, Australia; 4 Sydney Adventist Hospital, Sydney, Australia; 5 Save Sight Institute, University of Sydney, Sydney, Australia

**Keywords:** Cataract surgery, Refraction, Phacoemulsification

## Abstract

**Purpose:**

After cataract phacoemulsification surgery, spherical equivalent refraction (SER) may be affected by factors including corneal curvature, effective lens position and axial length. While refractive outcomes have been assessed in the immediate post-operative period, longer-term changes in refraction have not been reported. The purpose of this study was to investigate the timeline changes in refraction after cataract surgery over a period of 3 years.

**Methods:**

This was a retrospective observational study that included 344 eyes of 204 patients who underwent cataract emulsification surgery between 1 January and 31 December 2018 at two private hospitals. Keratometry, anterior chamber depth (ACD), central corneal thickness (CCT) and axial length were measured at baseline and post-operatively at 1 month, 1 year, 2 years and 3 years. Changes in SER and ocular parameters were assessed at each post-operative timepoint.

**Results:**

Between 1 month and 3 years post-operatively, an overall myopic shift (0.32 ± 0.21 D, *p* < 0.001) occurred in 33.6% of eyes and a hypermetropic shift in 45.2% of eyes (0.35 ± 0.22 D, *p* < 0.001). In 21.2% of eyes, there was no reported change in SER between 1 month and 3 years. Significant changes in ACD (*p* = 0.04) and CCT (*p* < 0.001) occurred during the first year after surgery.

**Conclusion:**

The 3-year timeline changes in SER after cataract surgery were evaluated. As hypermetropic shift was the most common refractive change observed, it may be beneficial to aim for a more myopic post-operative refraction target. Patients should be advised of the potential for refractive changes after surgery.

## Introduction

Cataract is the leading cause of blindness worldwide, for which surgery is the mainstay of therapy [1]. Cataract surgery is the most common elective procedure globally, with demand projected to increase in line with an ageing population [2]. While cataract surgery aims to treat vision-related impairment by improving visual acuity, it also enables patients to achieve a post-operative refraction that is optimal for activities of daily living [3].

In the current literature, after phacoemulsification and intraocular lens (IOL) insertion, successful refraction is defined as ± 1.00 dioptre (D) of target spherical equivalent refraction (SER) [4]. There is much variation in clinical practice regarding what is considered the ideal post-operative SER, allowing for optimal uncorrected distance and near vision. Generally, target SER has been reported to lie between − 0.50 and − 1.50 D [5, 6]. Although several studies have identified the proportion of patients achieving target post-operative SER [3, 4], there are limited data detailing the changes in refraction after cataract surgery in the long term [5]. In addition, there is a paucity of data on refractive changes obtained in a contemporary cohort and extending beyond 4 weeks post-operatively.

Post-operative refraction may be influenced by several factors including corneal curvature, axial length (AL) and effective lens position (ELP). While the impact of changes in AL and corneal curvature on SER is not well established [7], post-operative anterior chamber depth (ACD), used as an indicator of ELP, has been shown to account for a significant proportion of refractive error [8, 9]. A decrease in ACD is associated with myopic shift, while an increase leads to hypermetropic shift [10, 11].

The purpose of this study was to identify the timeline changes in post-operative ocular parameters and SER over a period of 36 months. Understanding these changes will facilitate the prediction and correction of refractive error in the long term after cataract surgery.

## Materials and methods

### Study population

This retrospective observational study included 344 eyes of 204 patients who underwent cataract phacoemulsification surgery at two private hospitals from 1 January 2018 to 31 December 2018. All patients across both sites were operated on by a single ophthalmic surgeon and had cataract phacoemulsification using Alcon Constellation OZil IP system and 0.9-mm mini-flared 45-degree Kelman phaco tip through a 2.75-mm temporal corneal incision. All patients received Alcon SN60WF IOL. Patients who had a superior corneal incision or had cataract surgery combined with another procedure were excluded. Patients who did not achieve a post-operative visual acuity of 6/12 or better were also excluded.

### Data collection

Demographic data were collected on patient age, defined as age at time of surgery, and sex. All participants underwent a comprehensive ophthalmic examination 1 week prior to surgery and at the following timepoints post-operatively: 1 month, 1 year, 2 years and 3 years. Keratometry, ACD, central corneal thickness (CCT) and AL were measured using IOL Master 700 (Carl Zeiss, Germany) at each timepoint. Keratometry was recorded in two meridians, the flat meridian of the anterior corneal surface (K1) and the steep meridian of the anterior corneal surface (K2). The IOL for each patient was selected based on the average of the SRK/T, Haigis, Holladay 2 and Barrett Universal II IOL formulae. Refractive power was assessed preliminarily using the Topcon KR-800 Auto Kerato-Refractometer (Topcon Co. Tokyo, Japan). Patients then had SER assessed using subjective refraction. SER was calculated as the sum of the sphere power and one-half of the cylinder power. Target SER was defined as between − 0.50 and − 1.50 D. All assessments and measurements were conducted by the same ophthalmic surgeon.

### Statistical analysis

Data were analysed using IBM SPSS Statistics, version 26.0 (IBM Corp, Armonk, NY). Descriptive statistics were used for patient demographic data. The changes in SER and ocular parameters K1, K2, ACD, CCT and AL were evaluated over each 12-month period during the 3 years of follow-up. Changes were calculated as mean ± standard deviation. The paired t-test was used to compare the results of the same patient at different timepoints post-operatively.

### Ethical approval

This study was approved by the Human Research Ethics Committee of the Northern Sydney Local Health District and was conducted in accordance with the tenets of the Declaration of Helsinki. Informed consent was obtained from all participants.

## Results

A total of 344 eyes (177 left eyes, 167 right eyes) of 204 patients were included in this study. Mean participant age was 68.17 ± 7.15 years. Of the eyes that received cataract surgery, 199 (57.8%) were of female patients and 145 (42.2%) were of male patients. The mean IOL used was 19.44 ± 4.14 D. Baseline patient demographics and pre-operative characteristics are shown in Table 1.

**Table 1:** Baseline patient characteristics

Number of patients/eyes	204/344
Age (years); [mean ± SD (range)]	68.17 ± 7.15 years (37–90)
Sex [*n*, (%)]	
Male	145 (42.2)
Female	199 (57.8)
Eye laterality [*n*, (%)]	
Right	167 (48.5)
Left	177 (51.5)
K1 (D); [mean ± SD (range)]	43.61 ± 1.37 (38.43–46.58)
K2 (D); [mean ± SD (range)]	44.44 ± 1.37 (40.09–48.89)
ACD (mm); [mean ± SD (range)]	3.09 ± 0.37 (2.25–4.26)
CCT (μm); [mean ± SD (range)]	545.76 ± 33.65 (463.10–667.20)
AL (mm); [mean ± SD (range)]	24.41 ± 1.63 (20.39–33.53)
IOL (D); [mean ± SD (range)]	19.44 ± 4.14 (6–29.5)

*SD* standard deviation, *ACD* anterior chamber depth, *CCT* central corneal thickness, *AL* axial length, *IOL* intraocular lens

The proportion of eyes achieving target refraction during the 3 years of follow-up were 83.5% at 1 month, 92.3% at 1 year, 82.7% at 2 years and 81.9% at 3 years. At 3 years, 61.9% of patients achieved SER between − 0.25 D and − 1.0 D. Compared to 1 month after cataract surgery, at 3 years post-operatively, an overall myopic shift (0.32 ± 0.21 D, *p* < 0.001) occurred in 33.6% of eyes, a hypermetropic shift occurred in 45.2% of eyes (0.35 ± 0.22 D, *p* < 0.001), and 21.2% of eyes recorded no change in SER.

**Fig. 1:**
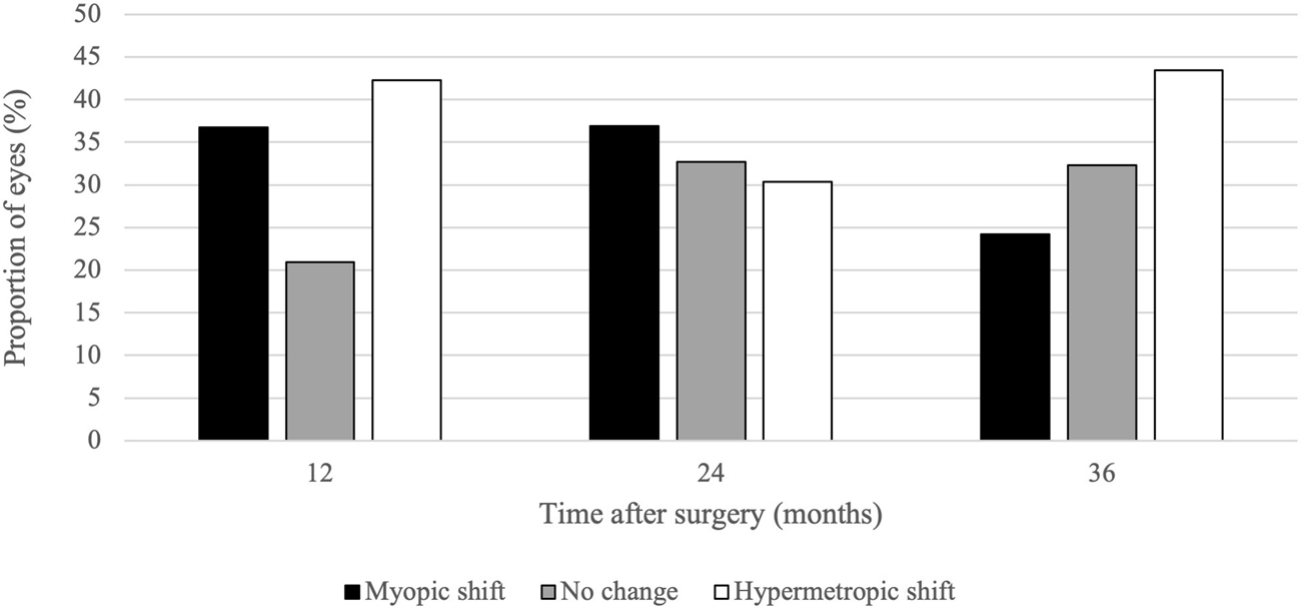
Direction of 12-month changes in SER after cataract surgery. *SER* spherical equivalent refraction

Shifts in refraction were assessed over each 12-month period of follow-up. The direction of SER changes is presented in Fig. 1. During the first 12 months, 36.6% of eyes underwent myopic shift (*p* < 0.001) and 42.1% underwent hypermetropic shift (*p* < 0.001). Between 1 and 2 years, myopic shift occurred in 36.9% (*p* = 0.22) and hypermetropic shift occurred in 30.4% of eyes (*p* = 0.016). In the final 12 months of follow-up, 24.2% of eyes become more myopic (*p* = 0.59) and 43.4% of eyes became more hypermetropic (*p* = 0.38); however, these changes were not significant.

**Fig. 2:**
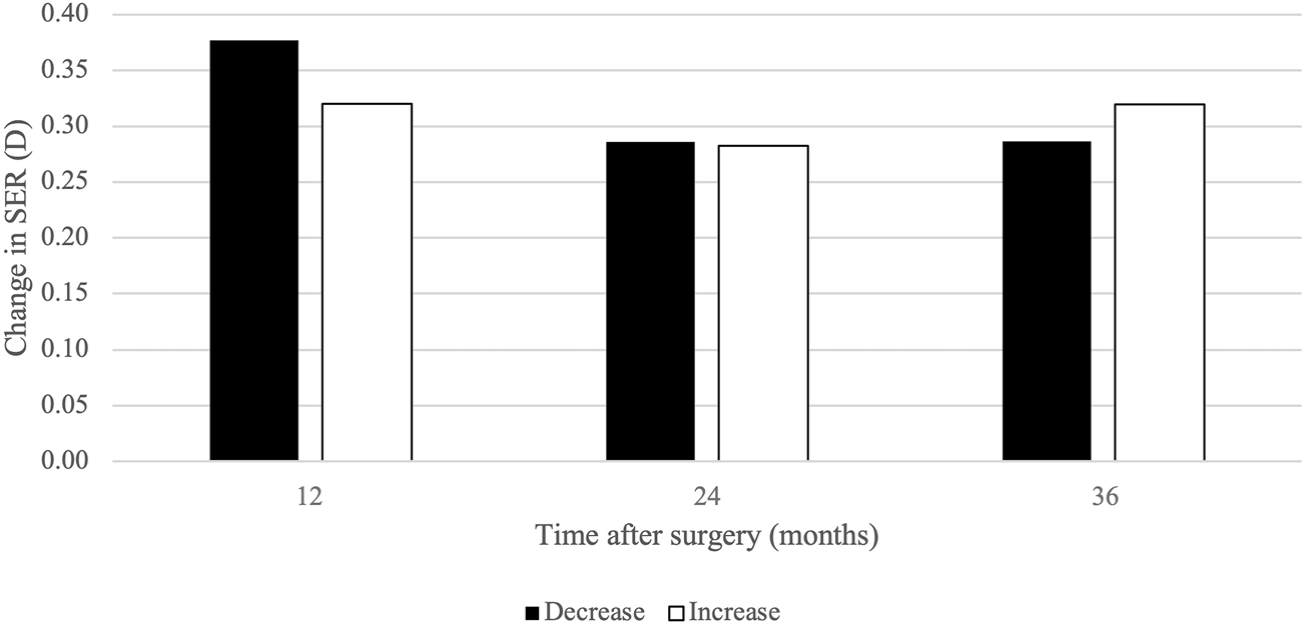
Mean 12-month changes in SER after cataract surgery. *SER* spherical equivalent refraction

The mean 12-month changes in SER are shown in Fig. 2. The greatest changes occurred in the first 12 months after surgery, with a decrease of 0.38 ± 0.28 D in eyes with myopic shift and an increase of 0.32 ± 0.29 D in eyes with hypermetropic shift. During the second 12-month period after surgery, the mean myopic change was 0.29 ± 0.16 D and the mean hypermetropic change was 0.28 ± 0.14 D. In the final 12-month period of follow-up, the myopic and hypermetropic changes were 0.29 ± 0.14 D and 0.32 ± 0.18 D, respectively.

The timeline changes in keratometry were also assessed. Although mean K1 demonstrated variability during the 3 years of follow-up, these changes were only significant during the first 12 months of follow-up (*p* = 0.003) and were not significant between 1 and 2 years (*p* = 0.27) and between 2 and 3 years (*p* = 0.15). There were no significant changes to K2 during the study period.

**Fig. 3:**
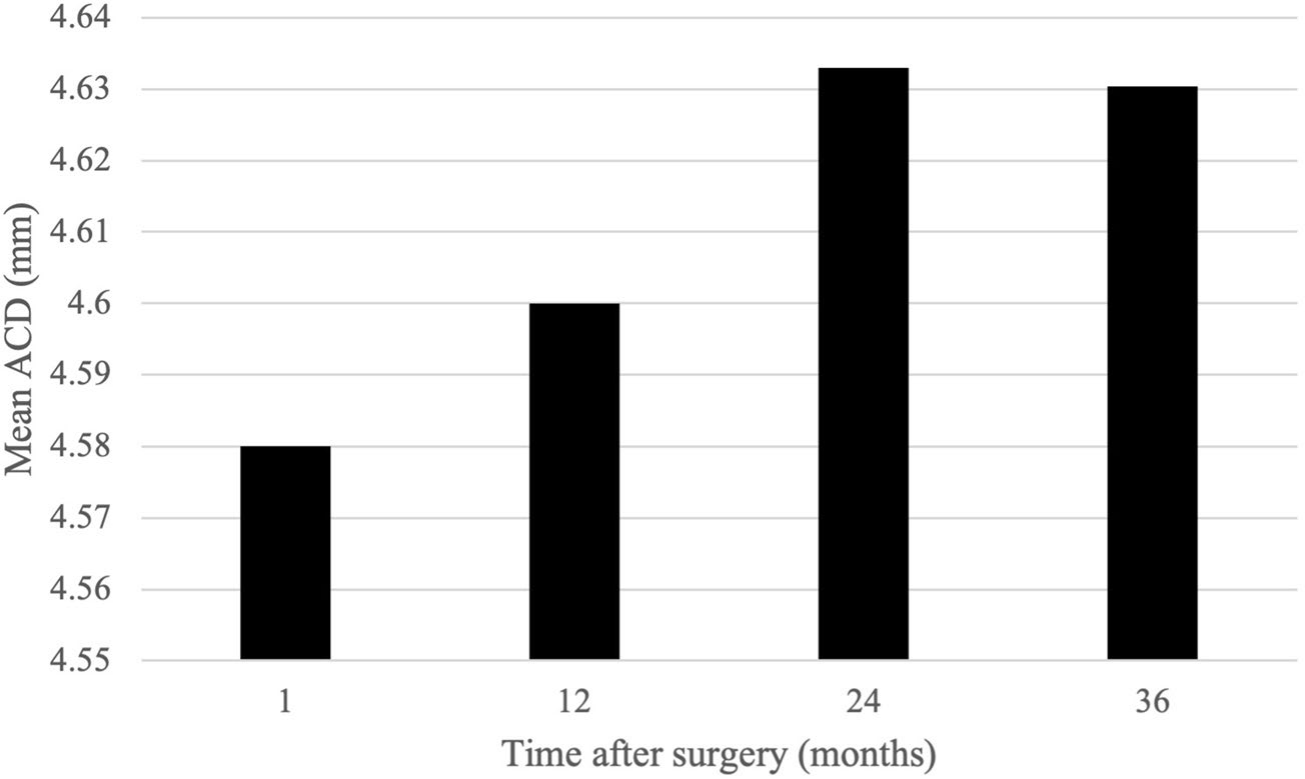
Mean ACD after cataract surgery. *ACD* anterior chamber depth

Mean ACD evaluated during the 3 years of follow-up is shown in Fig. 3. Significant increases in ACD occurred between 1 month and 1 year (*p* = 0.04) and between 1 and 2 years (*p* = 0.007) after surgery. There were no significant changes between 2 and 3 years post-operatively (*p* = 0.22). At the end of 3 years, the increase in ACD was greater in eyes that demonstrated hypermetropic shift (0.067 ± 0.14 mm) compared to eyes that demonstrated myopic shift (0.036 ± 0.16 mm).

**Fig. 4:**
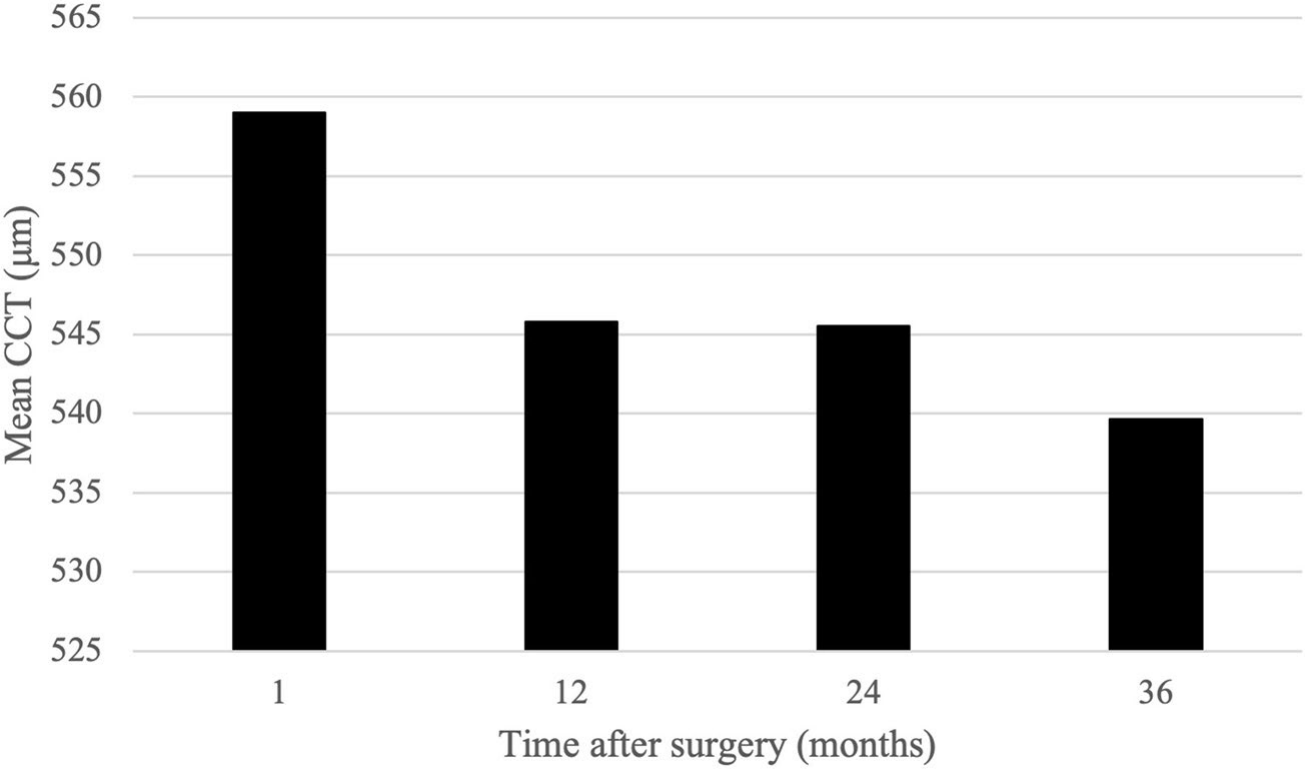
Mean CCT after cataract surgery. *CCT* central corneal thickness

CCT was similarly evaluated (Fig. 4), revealing significant changes in CCT between 1 month and 1 year (*p* < 0.001). Changes in CCT were not significant between 1 and 2 years (*p* = 0.090) and between 2 and 3 years (*p* = 0.059) post-operatively. Throughout the study period, there were no significant changes in AL.

## Discussion

To the best of our knowledge, this study is the first to present the 3-year timeline changes in refraction after cataract phacoemulsification surgery. Our findings suggest that significant changes in SER occur during the first 2 years after cataract surgery, with the greatest degree of change observed in the first 12 months post-operatively. The changes in SER observed may be due to a combination of ACD deepening and variations in corneal curvature. At the end of the study period, the most common overall direction of change was hypermetropic shift, observed in nearly half of participants.

Existing literature has primarily investigated cataract surgery outcomes by exploring differences between pre-operative and post-operative refraction [5, 12], or by examining discrepancies between predicted and actual outcomes [3, 4, 13–15]. Few have detailed the direction and extent of refractive shifts occurring post-operatively. In addition, studies assessing refraction after cataract surgery have been restricted to short follow-up periods ranging from 4 weeks [2] to at most 12 months [16], after which much regarding refractive outcomes is unknown.

Strengths of the current study include the regularity and duration of patient follow-up, allowing for long-term outcomes to be analysed and compared at 12-month intervals. Consequently, our results contrast with other reports of post-operative refractive stability occurring much earlier after surgery [10, 17]. As all procedures were performed by a single surgeon, operation-dependent factors influencing refraction were minimised, such as variability in size and position of the capsulorhexis [18]. Target refraction, post-operative dates, and ocular measurements including keratometry and axial length were recorded for all patients, unlike other studies where such data were unavailable [5, 16]. Furthermore, as opposed to autorefraction alone [19, 20], subjective refraction was used for final refractive testing, which is considered gold standard for refractive error assessment [21].

One of the biometric factors shown to influence post-operative refraction is ACD, which has been used as an approximation of ELP [9, 22]. The post-operative position of the implanted IOL, or ELP, is a key parameter affecting SER after cataract surgery. Methods to improve the prediction of ELP have been proposed, and however, inaccurate ELP determination remains a significant cause of refractive surprise and total SER prediction error [9]. As post-operative ACD can be used as an indicator of post-operative ELP, modern formulae such as the Holladay II, Haigis and Barrett Universal II incorporate pre-operative ACD into IOL power calculations [9, 10]. In concordance with other studies [10, 23], we found statistically significant increases in mean ACD post-operatively. Posterior movement of the IOL, consequently altering the ELP, has been reported in the first 12 months after cataract surgery [16], with axial position and stability of the IOL affected by processes such as capsular bag fibrosis and contraction [24]. The changes in ACD we observed may contribute to the shifts in refraction reported during the 3 years post-operatively, particularly as deepening of the anterior chamber was greater in eyes that underwent hypermetropic shift [10].

The impact of corneal curvature on post-operative refractive outcomes was also considered. Existing studies have not found significant differences between keratometric values after cataract surgery and, however, have only recorded changes in mean corneal curvature [16, 19]. We reported significant changes in K1 between 1 month and 1 year post-operatively. These alterations in corneal curvature have been partly attributed to corneal steepening as a consequence of corneal oedema [20, 25]. After the initial post-procedural period, continued refractive shift may be due to natural variations in corneal curvature, with reported differences of up to 0.50 D between measurements irrespective of operative status [26].

The second corneal parameter we evaluated was CCT, which demonstrated a mean decrease during the post-operative period. This is in agreement with other accounts of reduced corneal thickness after cataract surgery, although we do not expect long-term impacts of decreased CCT on refraction after the resolution of corneal oedema [19, 27]. Likewise to previous studies [7], there were no significant changes in AL throughout patient follow-up.

Limitations of our study include the potential for inter-eye correlation bias, as both eyes from patients who received bilateral cataract surgery during the study period were analysed. Another limitation is the unavailability of data on patients who developed posterior capsule opacification (PCO), which is an important post-operative complication that may result in bias, as it affects visual function and assessment of refraction [28]. As our study is retrospective in nature, future prospective studies are required to validate findings. Studies examining outcomes in patients with toric and multifocal IOLs will also be useful. Furthermore, as all procedures were performed by a single surgeon in a private hospital setting, the sample size was relatively small, and the applicability of results may be limited to cohorts undergoing uncomplicated surgery in similar settings.

## Conclusion

This study is the first to recognise the 3-year timeline changes in refraction after cataract surgery. As hypermetropic shift was the most common direction of change observed over the study period, it may be beneficial to aim for a more myopic refractive target during IOL selection. Refractive changes should also be considered during patient discussion of expectations and outcomes after surgery. Further studies on long-term refractive outcomes are needed.
